# Development of a histamine aptasensor for food safety monitoring

**DOI:** 10.1038/s41598-019-52876-1

**Published:** 2019-11-13

**Authors:** Mohammed Dwidar, Yohei Yokobayashi

**Affiliations:** 1 Nucleic Acid Chemistry and Engineering Unit, Okinawa Institute of Science and Technology Graduate University, Onna, Okinawa 904-0495 Japan; 2 Present Address: Center for Microbiome and Human Health, Lerner Research Institute, Cleveland Clinic, Cleveland, OH 44195 USA

**Keywords:** Bioanalytical chemistry, Nucleic acids, Assay systems

## Abstract

Histamine produced by bacteria through decarboxylation of histidine in spoiled foods such as fish is known to cause food poisoning. Therefore, accurate and facile measurement of histamine is of practical importance. Using the recently discovered RNA aptamer that specifically recognizes histamine (A1-949 aptamer), we developed an aptasensor based on the structure-switching mechanism. Specifically, the aptamer A1-949 was fluorescently labeled at the 5′ end and hybridized with a short quencher DNA strand that is partially complementary to the aptamer. The quencher strand was modified with a fluorescence quencher at its 3′ terminus. Displacement of the quencher strand upon histamine binding results in an increased fluorescence. After optimizing the assay condition, the enantiomeric version of the aptasensor (L-RNA and L-DNA) was synthesized which could detect the achiral analyte with identical sensitivity and improved biochemical stability. The aptasensor performance was validated by measuring fish samples spiked with known concentrations of histamine. Finally, histamine content in spoiled fish samples was measured, and the results were compared with the measurements using a commercial enzymatic assay kit.

## Introduction

Histamine is an important metabolite involved in a number of biological processes. For example, if improperly handled, histamine can accumulate in fish and dairy products containing bacteria that can decarboxylate histidine to produce histamine. Because histamine is heat resistant, its concentration does not decrease significantly or can even increase during cooking^1^. Histamine levels in food products especially fish are monitored routinely in the food industry to prevent food poisoning which can occur with histamine concentrations above 500 mg per kg of fish (~4.5 mM)^2^. FDA and European food regulations require that histamine concentrations in the fish should not exceed 50 mg/kg and 100 mg/kg, respectively^3^. Histamine released from basophils isolated from allergy patients is also used for diagnostic purposes (histamine release test)^4^. High level of histamine in plasma and urine is an important indicator of anaphylactic shock^5^. Furthermore, measurement of histamine released by cultured cells can benefit research in immunology and neuroscience^6,7^.

Currently, the most popular commercially available assays for quantifying histamine in biological samples are based on enzyme-linked immunosorbent assay (ELISA) or colorimetric enzymatic assay using histamine dehydrogenase with approximate detection limits of 0.1 µM and 0.5 µM, respectively^8–10^. Although these methods based on antibodies and enzymes are well established, dependence on recombinant proteins makes these conventional assays costly and require careful storage and handling. Instrumental analysis using high-performance liquid chromatography (HPLC)^11^, mass spectrometry^12^, and surface enhanced Raman spectroscopy (SERS)^13^ can offer advantages such as high sensitivity, but such assays are time consuming and require sophisticated instruments and skilled operators. Several small molecular probes for histamine have been reported but their applications have been limited^7,14^.

Aptamers are single stranded oligonucleotides (DNA or RNA) capable of molecular recognition. Aptamers are usually selected *in vitro* from a pool of 10^14^ to 10^15^ random sequences through a process called systematic evolution of ligands by exponential enrichment (SELEX)^15^. The target can be a small molecule, a protein, or even a whole cell. As molecular recognition elements, aptamers offer several advantages over antibodies^16^. For example, aptamers can be chemically synthesized and thus are less costly to produce and more stable compared to antibodies. Moreover, the predictable nature of nucleic acid hybridization has facilitated the development of numerous strategies to engineer aptamers into sensors (aptasensors)^17^.

**Figure 1:**
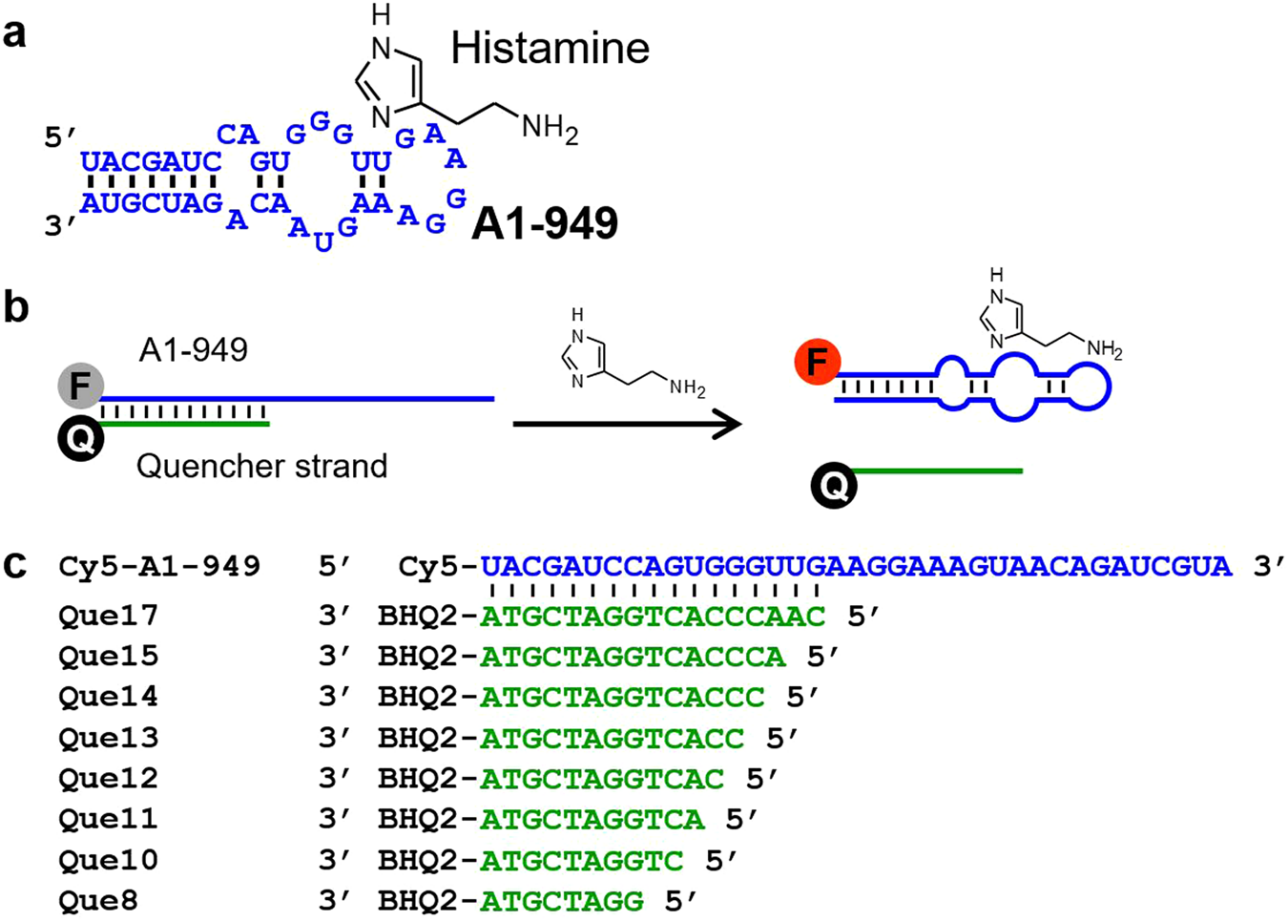
Histamine aptamer and aptasensor. (**a**) A1-949 aptamer sequence and the secondary structure predicted by Mfold. (**b**) Schematic illustration of the histamine aptasensor design. The histamine aptamer (blue) is fluorescently labeled at the 5′ end, and it is hybridized with a quencher DNA strand. Binding of histamine to the aptamer displaces the quencher strand resulting in an increased fluorescence. (**c**) BHQ2-modified quencher strands tested in this study.

On the other hand, development of aptamers that recognize molecules of practical importance still limit broader applications of aptamers. In a previous study, we successfully selected an RNA aptamer (A1-949 aptamer) (Fig. 1a) that can bind histamine with high affinity and specificity from a pool of 3 × 10^14^ random sequences through SELEX aided by deep sequencing^18^. In the current study, we employed the A1-949 aptamer to engineer a fluorescence-based aptasensor that can detect histamine concentration as low as 1 μM.

## Results and Discussion

### Optimization of histamine aptasensor based on structure-switching mechanism

The recently discovered histamine aptamer A1-949 (Fig. 1a) binds histamine with a dissociation constant (K_d_) of 370 nM while associating with L-histidine with K_d_ > 20 μM based on isothermal titration calorimetry (ITC)^18^. Based on the putative secondary structure suggested by Mfold^19^, structure-switching aptasensors were designed as illustrated in Fig. 1b ^20,21^. Briefly, the A1-949 aptamer was chemically synthesized with a Cy5 fluorophore at the 5′ terminus (Cy5-A1-949) (Fig. 1c). DNA sequences that are partially complementary to the 5′ region of A1-949 of varying lengths were synthesized with a quencher (BHQ2) on the 3′ terminus (Que*N*: *N* = 8, 10–15, 17) (Fig. 1c). These quencher strands quench Cy5 fluorescence when hybridized with Cy5-A1-949. When histamine binds to Cy5-A1-949, the quencher strand is displaced, resulting in activation of Cy5 fluorescence (Fig. 1b).

**Figure 2:**
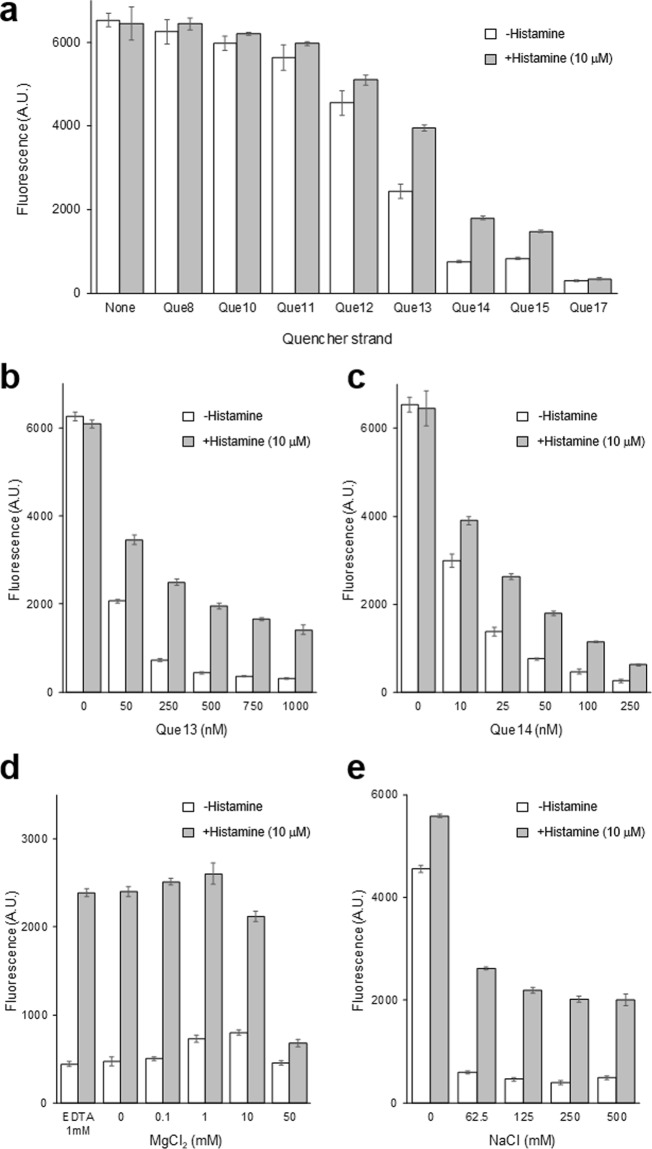
Optimization of the aptasensor design and assay conditions. Cy5-A1-949 concentration was 5 nM in all experiments shown. (**a**) Evaluation of the quencher strands depicted in Fig. 1C. Que*N* concentration was 50 nM. (**b**) Optimization of Que13 concentration. (**c**) Optimization of Que14 concentration. (**d**) Optimization of MgCl_2_ concentration with Que13 (500 nM) as the quencher strand. (**e**) Optimization of NaCl concentration with Que13 (500 nM) as the quencher strand. The reported values are averages of at least 3 technical replicates and the error bars show standard deviations.

First, the length of the quencher strand was varied from 8 to 17 nt. Cy5-A1-949 (5 nM) and an appropriate quencher strand (50 nM, Que*N*) were mixed with or without histamine (10 μM) in the initial assay buffer (50 mM HEPES, pH 7.0, 250 mM NaCl, 0.1 mM MgCl_2_, 0.01% Tween 20) and heated to 55 °C for 20 min. Cy5 fluorescence was measured after cooling the mixture to room temperature. Que8, Que10, Que11, and Que12 showed high fluorescence comparable to Cy5-A1-949 alone, indicating that these quencher oligos do not hybridize strongly with the aptamer (Fig. 2a). Low fluorescence was observed with Que17 with or without histamine, suggesting that this quencher strand is strongly hybridized with Cy5-A1-949 and cannot be displaced by histamine. However, Que13 and Que14 allowed moderate fluorescence increases in the presence of 10 μM histamine, up to 1.6- and 2.4-fold, respectively, (Fig. 2a). Therefore, we then varied Que13 (Fig. 2b) and Que14 (Fig. 2c) concentrations. We observed that 500 nM Que13 results in an optimal 4.5-fold increase in fluorescence in the presence of 10 μM histamine (Fig. 2b).

Next, we varied the MgCl_2_ concentration and discovered that the aptasensor was not dependent on MgCl_2_. In fact, high Mg^2+^ concentrations degraded the sensor performance (Fig. 2d). We observed no significant change in the sensor response in the NaCl concentration range 125–500 mM (Fig. 2e). Based on these results, we determined the optimal assay conditions as follows: Cy5-A1-949 (5 nM), Que13 (500 nM), HEPES (50 mM, pH 7.0), NaCl (250 mM), EDTA (1 mM), and Tween-20 (0.01%).

### Aptasensor specificity and sensitivity

**Figure 3:**
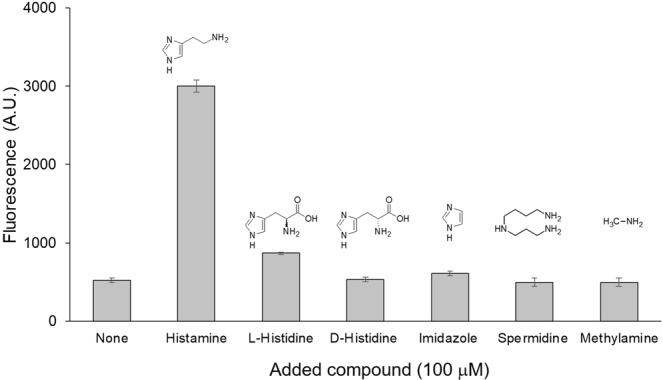
Specificity of the aptasensor. Responses of the aptasensors to histamine, L-histidine, D-histidine, imidazole, spermidine, and methylamine were measured at 100 μM. The reported values are averages of at least 3 technical replicates and the error bars show standard deviations.

**Figure 4:**
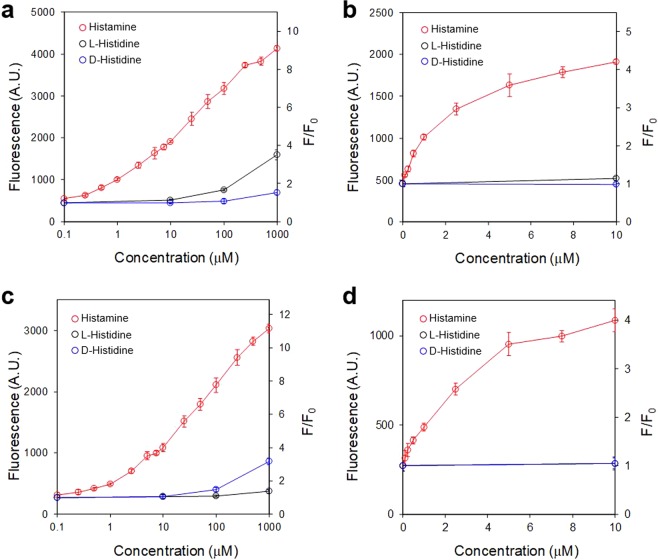
Dose-dependent responses of the aptasensors to histamine and histidine stereoisomers. Aptasensor Cy5-A1-949/Que13 in (**a**) semi-log plot and (**b**) linear plot at the low concentration range. Mirror-image aptasensor Cy3-A1-949*/Que13* in (**c**) semi-log plot and (**d**) linear plot at the low concentration range. The reported values are averages of at least 3 technical replicates and the error bars show standard deviations.

Specificity of the aptasensor under the optimized assay conditions was evaluated by observing its responses to a range of biochemically relevant compounds. D-histidine, imidazole, spermidine, and methylamine did not show detectable fluorescence increase at 100 µM while L-histidine showed a modest increase (1.7-fold) (Fig. 3). The sensor performance was further evaluated in the presence of varying concentrations of histamine and histidine stereoisomers (Fig. 4a,b). The aptasensor could detect histamine concentration as low as 1 μM. Although the aptasensor displayed an appreciable response at 1000 μM L-histidine, the signal was lower than that of histamine at 5 μM. Moreover, the aptasensor showed negligible response to D-histidine even at 1000 μM (Fig. 4a).

### Mirror-image aptasensor displays improved selectivity against L-histidine

RNA is highly susceptible to ribonucleases which are widely found in environmental and biological samples. Therefore, aptasensors based on RNA aptamers are limited in their practical applications. Since histamine is achiral, however, an aptamer entirely composed of L-ribonucleotides should display an identical affinity to histamine. Such L-RNA aptamers and aptasensors have been shown to be highly resistant to nucleases and stable in biological samples^22–25^. Therefore, the A1-949 aptamer sequence was synthesized using L-ribonucleotides modified with a Cy3 fluorophore at the 5′ end (Cy3-A1-949*). Corresponding quencher DNA (Que13*) was also synthesized using L-deoxyribonucleotides with a BHQ2 modification at the 3′ terminus. The mirror-image (L-) aptasensor was evaluated using histamine and histidine stereoisomers (Fig. 4c,d). As expected, the response to histamine by the L-aptasensor was comparable to that of the original sensor. More importantly, the L-aptasensor showed negligible response to L-histidine as expected from the inversion of the sensor’s stereochemistry. The improved selectivity of the L-aptasensor against the proteinogenic L-histidine further benefits applications of the sensor for the food industry (Fig. 4c).

### Quantification of histamine in spiked fish samples

To demonstrate the performance of the L-aptasensor in a practical setting, we used the sensor to measure fish samples spiked with known concentrations of histamine. We obtained fresh tuna meat which is frequently associated with histaminosis and routinely screened for possible histamine contamination^2^. Raw tuna pieces were spiked with various histamine concentrations, heated, homogenized, and processed by ultrafiltration to remove macromolecules. The extracted histamine samples were assayed by the L-aptasensor as described above. The results indicate that the aptasensor shows a good response to the histamine-spiked tuna extract (Table 1). Moreover, we measured naturally produced histamine in spoiled tuna samples using the L-aptasensor and a commercial histamine assay kit based on histamine dehydrogenase. The two methods yielded comparable results (Table 2).

**Table 1:** Recovery of histamine spiked in fresh tuna samples.

Histamine spiked (μmol/kg)	Recovered concentration (μmol/kg)		% Recovered	
	Sample 1	Sample 2	Sample 1	Sample 2
0	16 ± 1	17 ± 1		
200	226 ± 45	218 ± 44	113 ± 23	109 ± 22
500	520 ± 27	502 ± 23	104 ± 5	100 ± 5
1000	1000 ± 112	1147 ± 56	100 ± 11	115 ± 6
2000	2085 ± 249	1985 ± 296	104 ± 12	99 ± 15
5000	4403 ± 566	4017 ± 461	88 ± 11	80 ± 9

Two tuna samples were measured at each spiked histamine concentration. The values are averages and standard deviations of 5 wells.

**Table 2:** Measurement of histamine in spoiled tuna by the L-aptasensor and a commercial enzymatic assay kit.

Four-day-old spoiled tuna meat	Histamine dehydrogenase kit (μmol/kg)	L-aptasensor (μmol/kg)
Sample 1	3902 ± 261	3888 ± 765
Sample 2	3906 ± 237	4021 ± 523

Two tuna samples were measured by the two methods. The numbers are averages and the standard deviations of 3 measurements for the histamine dehydrogenase kit, and 8 measurements for the L-aptasensor.

### Histamine aptamers and aptasensors

Selection of DNA histamine aptamers have been recently reported by Mairal Lerga *et al*.^26^. A notable difference in their selection method was the use of histamine immobilized via the amino group whereas we immobilized L-histidine leaving both the imidazole and the amine available for the aptamers to interact with. While the reported K_d_ values of 3~34 nM are impressive, these values were acquired by indirect binding assays using immobilized histamine as the binding target. The reported histamine assay was also based on competitive binding of the selected aptamer to the immobilized histamine in the presence of the analyte, followed by washing and enzymatic signal development. Our histamine aptasensor used the A1-949 aptamer whose binding with histamine and selectivity against L-histidine was directly characterized in solution by ITC^18^. In this work, we further demonstrated the specificity of the aptasensor against five additional compounds structurally related to histamine (Fig. 4). Consequently, it is highly likely that A1-949 and the aptasensor recognize both the imidazole and amine groups of histamine. The aptasensor based on the structure-switching mechanism also allows homogenous detection of histamine which is convenient in practical settings.

## Conclusion

We developed a robust histamine aptasensor based on the structure-switching mechanism. The achiral nature of histamine allowed us to use L-RNA and L-DNA strands to construct the aptasensor which resulted in enhanced biochemical stability and improved selectivity against L-histidine. The aptasensor could reliably detect histamine concentrations as low as 1 μM, and it was used to detect histamine in tuna samples.

## Methods

### Oligonucleotides

The histamine-binding RNA aptamer labeled with Cy5 at the 5′ end (Cy5-A1-949) and 3′ BHQ2-labeled DNAs (Que*N*: *N* = 8, 10–15, 17) were purchased from FASMAC and Macrogen, respectively (Fig. 1c). The 5′-Cy3-labeled RNA aptamer (Cy3-A1-949*) and 3′ BHQ2-labeled DNA (Que13*) in L-nucleotides were synthesized by GeneDesign.

### Aptasensor-based assay of histamine

Both the fluorescently labeled aptamer Cy5-A1-949 and the quencher DNA Que*N* were stored as 100 μM stocks at −80 °C. Unless otherwise indicated, Cy5-A1-949 was diluted to 5.5 nM and Que*N* was diluted to 550 nM in the appropriate assay buffer. Aliquots (90 μL) of Cy5-A1-949 and Que*N* solution were dispensed in the wells of a black non-binding 384-well microplate (Greiner) and the histamine samples (10 μL) prepared in the same buffer were added to each well. The plate was incubated at 55 °C for 20 min and then cooled at room temperature for 1 h. Subsequently, fluorescence was measured at 650 nm excitation and 670 nm emission using M1000 PRO microplate reader (Tecan). Measurements employing the L-enantiomer sensor oligonucleotides Cy3-A1-949* and Que13* were performed using a total assay volume of 50 μL instead of 100 μL, and with 550 nm excitation and 570 nm emission.

### Measurement of spiked histamine in tuna samples using the L-aptasensor

Fresh skinless pieces of tuna fish were purchased at a local market and stored at −20 °C until use. The meat was cut into ~0.5 g pieces, weighed precisely and kept in 5 mL Eppendorf tubes. Histamine was added at an appropriate concentration in 4.5 mL assay buffer (50 mM HEPES, pH 7.0, 250 mM NaCl, 1 mM EDTA). Two samples were prepared for each histamine concentration tested. The mixture in each tube was then homogenized, vortexed, and heated to 90 °C for 20 min. The samples were centrifuged and the supernatants were collected. The supernatants were then filtered through 1 kDa-cutoff ultrafiltration devices (PALL Microsep Advance Centrifugal Device, 1 K, 5 mL) to remove macromolecular components. Five microliters of the filtrate or the standard samples which were prepared in the same assay buffer, were then added to 45 μL of the L-form aptasensor solution (50 mM HEPES, pH 7.0, 250 mM NaCl, 1 mM EDTA, 0.01% Tween 20) in the assay wells as described above without further dilution. The standard curve was fitted to a cubic function (Supplementary Fig. S1) which was used to calculate the histamine concentrations of the spiked samples.

### Measurement of histamine in 4-day old spoiled tuna samples

Approximately 1.5~2.0 g pieces of tuna fish meat were incubated at 30 °C inside 15 mL tubes for 4 days. Each sample was then diluted with an equivalent volume (100 µL for every 100 mg of tuna) of the assay buffer (50 mM HEPES, pH 7.0, 250 mM NaCl, 1 mM EDTA), vortexed, homogenized, and processed as described above. Due to the high histamine concentration in these samples, they were diluted 50-fold and 100-fold, and 5 μL aliquots of these dilutions or the standard samples were added to 45 µL of the L-form aptasensor solution (50 mM HEPES, pH 7.0, 250 mM NaCl, 1 mM EDTA, 0.01% Tween 20) in the assay wells. The standard curve was fitted to a cubic function (Supplementary Fig. S1) which was used to calculate the histamine concentrations of the tuna samples. Similarly, 5- and 10-fold sample dilutions were used to measure the histamine content using a commercial histamine-dehydrogenase based kit (BioAssay Systems, EnzyChrom Histamine Assay Kit) according to the manufacturer’s instructions.

## Supplementary information

Supplementary Information

## References

[1] Chung BY (2017). Effect of Different Cooking Methods on Histamine Levels in Selected Foods. Ann. Dermatol..

[2] Feng C, Teuber S, Gershwin ME (2016). Histamine (Scombroid) Fish Poisoning: a Comprehensive Review. Clin. Rev. Allergy Immunol..

[3] Yesudhason P (2013). Histamine levels in commercially important fresh and processed fish of Oman with reference to international standards. Food Chem.

[4] Platzer MH, Grattan CE, Poulsen LK, Skov PS (2005). Validation of basophil histamine release against the autologous serum skin test and outcome of serum-induced basophil histamine release studies in a large population of chronic urticaria patients. Allergy.

[5] Laroche D, Vergnaud MC, Sillard B, Soufarapis H, Bricard H (1991). Biochemical markers of anaphylactoid reactions to drugs. Comparison of plasma histamine and tryptase. Anesthesiology.

[6] Hu J (2007). Wide distribution and subcellular localization of histamine in sympathetic nervous systems of different species. Neurosci. Res..

[7] Kielland N, Vendrell M, Lavilla R, Chang YT (2012). Imaging histamine in live basophils and macrophages with a fluorescent mesoionic acid fluoride. Chem. Commun..

[8] Muscarella M, Lo Magro S, Campaniello M, Armentano A, Stacchini P (2013). Survey of histamine levels in fresh fish and fish products collected in Puglia (Italy) by ELISA and HPLC with fluorimetric detection. Food Control.

[9] Pessatti TL, Fontana JD, Pessatti ML (2004). Spectrophotometric determination of histamine in fisheries using an enzyme immunoassay method. Methods Mol. Biol..

[10] Sato T, Horiuchi T, Nishimura I (2005). Simple and rapid determination of histamine in food using a new histamine dehydrogenase from Rhizobium sp. Anal. Biochem..

[11] Onal A (2007). A review: Current analytical methods for the determination of biogenic amines in foods. Food Chem..

[12] Nei D, Nakamura N, Ishihara K, Kimura M, Satomi M (2017). A rapid screening of histamine concentration in fish fillet by direct analysis in real time mass spectrometry (DART-MS). Food Control.

[13] Janci T (2017). Determination of histamine in fish by Surface Enhanced Raman Spectroscopy using silver colloid SERS substrates. Food Chem..

[14] Seto D, Soh N, Nakano K, Imato T (2010). An amphiphilic fluorescent probe for the visualization of histamine in living cells. Bioorg. Med. Chem. Lett..

[15] Ruscito A, DeRosa MC (2016). Small-Molecule Binding Aptamers: Selection Strategies, Characterization, and Applications. Front. Chem..

[16] McKeague M, Derosa MC (2012). Challenges and opportunities for small molecule aptamer development. J. Nucleic Acids.

[17] Pfeiffer F, Mayer G (2016). Selection and Biosensor Application of Aptamers for Small Molecules. Front. Chem..

[18] Dwidar M (2019). Programmable Artificial Cells Using Histamine-Responsive Synthetic Riboswitch. J. Am. Chem. Soc..

[19] Zuker M (2003). Mfold web server for nucleic acid folding and hybridization prediction. Nucleic Acids Res..

[20] Feagin TA, Maganzini N, Soh HT (2018). Strategies for Creating Structure-Switching Aptamers. ACS Sens..

[21] Nutiu R, Li YF (2004). Structure-switching signaling aptamers: Transducing molecular recognition into fluorescence signaling. Chem. Eur. J..

[22] Chen H (2017). Generation of Biostable L-aptamers against Achiral Targets by Chiral Inversion of Existing D-aptamers. Talanta.

[23] Luo X (2019). Exploiting the application of l-aptamer with excellent stability: an efficient sensing platform for malachite green in fish samples. Analyst.

[24] Olea C, Horning DP, Joyce GF (2012). Ligand-dependent exponential amplification of a self-replicating L-RNA enzyme. J. Am. Chem. Soc..

[25] Zhong W, Sczepanski JT (2019). A Mirror Image Fluorogenic Aptamer Sensor for Live-Cell Imaging of MicroRNAs. ACS Sens..

[26] Mairal Lerga T (2019). High Affinity Aptamer for the Detection of the Biogenic Amine Histamine. Anal. Chem..

