# Supplementary material for: Development of a histamine aptasensor for food safety monitoring
Source: Sci Rep. 2019 Nov 13;9:16659. doi: 10.1038/s41598-019-52876-1 (PMC6853955; doi:10.1038/s41598-019-52876-1)
Supplement: Supplementary file 1 — Supplementary Information [file 41598_2019_52876_MOESM1_ESM.pdf]

Supplementary Information

Development of a histamine aptasensor for food safety monitoring

Mohammed Dwidar and Yohei Yokobayashi*

Nucleic Acid Chemistry and Engineering Unit, Okinawa Institute of Science and Technology Graduate University, Onna, Okinawa, 904-0495, Japan

*Email: yohei.yokobayashi@oist.jp

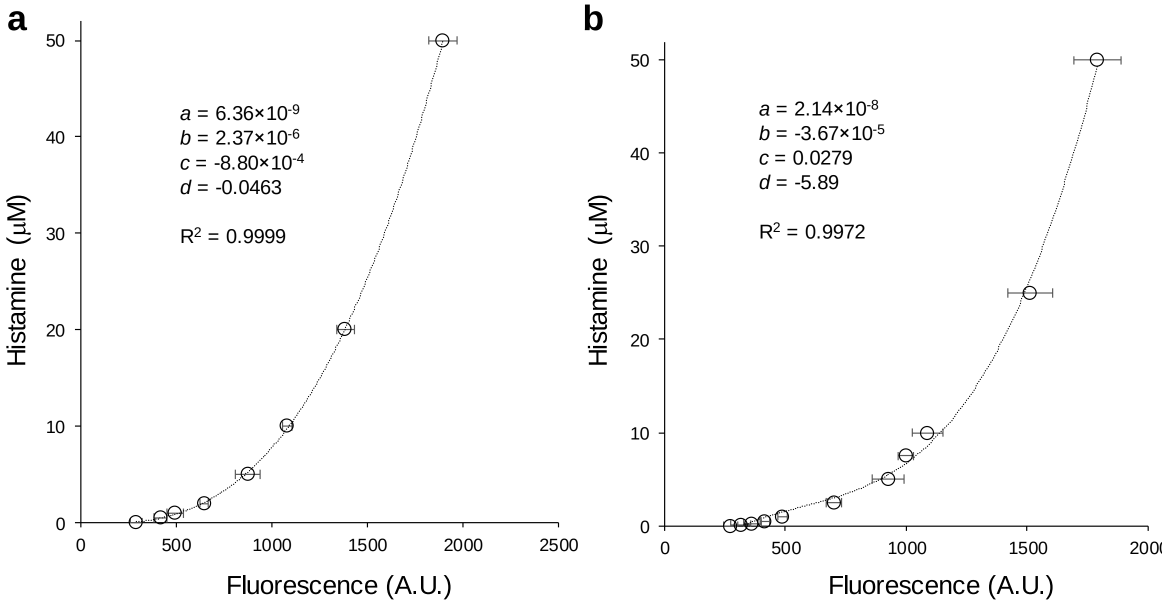

Figure S1 Standard curves of the histamine aptasensor. Each data point represents an average of 4 or more wells. The error bars represent standard deviations. Concentration of the standard histamine samples (Y-axis) were plotted against the aptasensor fluorescence output (X-axis), and the data were fitted to a cubic function ($y=ax^3+bx^2+cx+d$) using Microsoft Excel. (**a**) Standard curve used for the histamine-spiked tuna samples shown in Table 1. (**b**) Standard curve used for the histamine measurement in the spoiled tuna samples shown in Table 2.
